# THE DREAM TOOLKIT: CO-DEVELOPED TRAINING AND RESOURCES TO PROMOTE WELL-BEING OF PERSONS WITH DEMENTIA

**DOI:** 10.1093/geroni/igad104.2418

**Published:** 2023-12-21

**Authors:** Shannon Freeman, Laura Middleton, Chelsea Pelletier, Kelly Skinner, Kayla Regan, Rachael Donnelly, Heather Keller, DREAM Research Team

**Affiliations:** University of Northern British Columbia, Prince George, British Columbia, Canada; University of Waterloo, Waterloo, Ontario, Canada; University of Northern British Columbia, Prince George, British Columbia, Canada; University of Waterloo, Waterloo, Ontario, Canada; University of Waterloo, Waterloo, Ontario, Canada; University of Waterloo, Waterloo, Ontario, Canada; University of Waterloo, Waterloo, Ontario, Canada; University of Waterloo, Waterloo, Ontario, Canada

## Abstract

Promoting wellbeing of persons with dementia is a priority. Engaging multi-perspective partners to co-develop interventions creates impactful solutions. We will describe the process, output, and lessons from the Dementia Resources for Eating, Activity, and Meaningful inclusion (DREAM) project, which co-developed tools/resources with persons with dementia, care partners, community service providers, health care professionals, and researchers. We aimed to increase supports for physical activity, healthy eating, and wellbeing of persons with dementia. Our process included: 1) Engaging/maintaining the DREAM Steering Team; 2) Setting/navigating ways of engagement; 3) Prioritizing audience and content of the toolkit; 4) Drafting content & format of toolkit; 5) Iterative co-development of tools/resources; 6) Usability testing; 7) Implementation and evaluation. In virtual meetings, the DREAM Steering Team confirmed toolkit audiences (primary: community service providers; secondary: persons with dementia and care partners) and discussed and evolved content areas. An environmental scan identified existing, high-quality resources aligned with content areas. The DREAM Steering Team alongside additional community partners and external contractors iteratively co-developed new resources/tools to meet gaps. The DREAM toolkit includes a website, seven learning modules about dementia, healthy eating, and physical activity, a learning manual, six videos, nine handouts, and four wallet cards (www.dementiawellness.ca). Co-development participants rated the virtual co-development process highly in relation to the principles and enablers of Authentic Partnership in a process evaluation. Through the co-developed DREAM toolkit, we anticipate community service providers will learn to provide inclusive wellness programs and services to benefit persons with dementia and their families.

